# Do Income and Employment Uncertainty Affect Couple Stability? Evidence for France During the COVID-19 Pandemic

**DOI:** 10.1007/s10680-023-09665-4

**Published:** 2023-07-03

**Authors:** Anna Barbuscia, Ariane Pailhé, Anne Solaz, Epicov team, Nathalie Bajos, Josiane Warszawski, Guillaume Bagein, François Beck, Emilie Counil, Florence Jusot, Nathalie Lydie, Claude Martin, Laurence Meyer, Ariane Pailhé, Philippe Raynaud, Alexandra Rouquette, Delphine Rahib, Patrick Sicard, Rémy Slama, Alexis Spire

**Affiliations:** 1 Universitad del Pais Vasco (EHU/UPV), Leioa, Spain; 2 Institut national d’études démographiques (INED), Aubervilliers, France

**Keywords:** Economic uncertainty, Couple separation, Covid-19 pandemic, Unemployment, Divorce, France

## Abstract

Economic uncertainty and family dynamics are strictly connected. The increasing uncertainty generated by the Covid-19 pandemic is thus likely to affect couple relationships and stability, with potential opposite effects. Using data from the nationally representative EPICOV survey, that followed individuals throughout the first year of pandemic in France, we examined separation rates and how these were associated with different measures of employment and income uncertainty, including both pre-pandemic conditions and changes occurred during and after the first lockdown in Spring 2020 in France. Our results show increased rates of separation, especially among younger people, during the 6 months after the first lockdown, and a return to rates more similar to those observed in usual times, afterwards. Individuals who were unemployed and had lower income before the beginning of the pandemic were more likely to separate soon after the lockdown, while changes in employment conditions due to the lockdown were not linked with a higher separation risk. The job protection and the income compensation provided by the French state, as well a less stigmatising effect of unemployment occurred during the covid crisis, may explain the absence of effect. Self-declared deterioration in financial condition, especially when declared by men, was associated with higher separation risk for the whole year of observation.

## Introduction

Economic downturn and the resulting uncertainty strongly affect family dynamics: fertility, living arrangements, union formation and divorce are impacted (Kreyenfeld et al., 2012; Comolli et al. 2021; Vignoli et al., 2020; Sobotka et al. 2011). The effects of the particularly sharp decline in activity due to the Covid-19 pandemic on family structure has thus become a crucial question for public debate as well as for the scientific community. A widespread drop in births was observed during the first months of pandemic, with a recovery afterwards in most countries (Sobotka et al., 2021; Papon & Beaumel, 2021). However, how the uncertainty generated by the Covid-19 pandemic and the related economic crisis might have affected couple relationships and dynamics has been scarcely examined so far.

The consequences of these uncertainties for couple stability might potentially go in opposite directions. On the one hand, feelings of uncertainty can generate stress and tensions between the members of a couple, which might affect marital relationship, as predicted by theories of family stress (Conger et al., 1990; Schmid et al., 2021). Hence, the great uncertainty about financial resources and/or employment opportunities that some individuals have faced since the beginning of the pandemic crisis might have been a source of stress with potential negative consequence on intra-family relationships. On the other hand, living in couple may represent a sort of insurance against risk, as it might reduce uncertainty and allow to face financial strains (Schoen et al., 2002). In addition, as suggested by theories of family resilience (Black & Lobo, 2008; Walsh, 2003), to face a moment of crisis can reinforce couple relationship (Peterson et al., 2011). Some couples might also have got closer together and more connected thanks to the opportunity to prioritize their relationships and settle into slower rhythms of family life (Settersen et al., 2020), as the lockdown provided an opportunity to enjoy more leisure time together.

The evidence about the effects of the pandemic on couple stability is still scarce and has provided inconclusive evidence. Early data suggested an increase in divorce in the city of Wuhan after the first lockdown (Prasso, 2020), but quantitative studies on Europe have not found an increase in divorce (Fallesen et al., 2021). In the US, fewer divorces were observed in the initial months of the pandemic (March, April, May 2020) than during the same months in 2018 and 2019 (Manning & Payne, 2021; Westrick-Payne et al., 2022). A deficit in divorces was also observed in Japan during the first state of emergency (Ghaznavi et al., 2022; Komura & Ogawa, 2022). Stack bureaucratic procedures to divorce or the impossibility to spend some time apart before taking official action might have brought some couples to delay divorce despite low quality of relationship (Lebow, 2020). Even less is known about couple stability in the months following the first general lockdowns, and about the mechanisms that may have been playing a role during this exceptional period.

In this study, we examined separation rates throughout the first year of pandemic crisis in France and investigated the role of income and employment uncertainty on the risk of separation. We consider that separation provides a more immediate and timing picture of family relations compared to divorce: because of the long times required to divorce, especially during lockdowns (Lebow, 2020), the effect of the pandemic on couple stability may have been stronger if de facto couple situation is considered. The French context is an interesting one to examine. First, the first lockdown in Spring 2020 represented a real shock on employment: among those in employment, one third totally or partially stopped working, and young people were especially affected (Givord & Silhol, 2020). In the following months, the situation returned more “normal”, and most individuals who had stopped could start working again, but the situation remained highly uncertain in terms of future employment conditions due to possible restrictions. The health crisis also led to a decline in income: the average wage fell by 4.9% in the private sector and situations of precariousness became more common (Insee, 2021). A quarter of households reported that their financial situation worsened during the first lockdown, and previous research has showed that this deterioration of financial situation had a particularly negative effect on family relationships (Pailhé et al., 2022). Even in case of wage stability, the exceptional situation created economic uncertainty about both future employment opportunities and financial resources for many workers. This generalized increase in income and employment uncertainty due the Covid-19 pandemic provides an excellent opportunity to further understand the link between uncertainty and family relationships. Second, despite global uncertainty, workers were protected through instruments such as partial activity, where both employees and enterprises whose activity was reduced or totally stopped received an allowance. Thanks to the implementation of such measures, as well as the generalization of telework where possible, the decline in employment rate was limited (−0.9% in 2020) relatively to the drop in activity, and job protection was guaranteed in most cases. Therefore, situations of employment uncertainty did not necessarily mean an increase in income uncertainty, which allows to consider the two kinds of uncertainty -income and employment related- separately.

To answer our question, we used data from the EPICOV survey, a large representative sample collected at the beginning of the Covid-19 crisis and followed over time. Thanks to the rich information about employment and financial situation in the EPICOV survey, we were able to examine, both separately and jointly, different aspects of employment and income uncertainty, and how these interact in their relationship with couple stability. Interestingly, we were able to examine separately separations that occurred during the first months of the pandemic and those that occurred in the following months. Because the kind and levels of uncertainty differed in the two periods of time, we could expect them to play differently on couple stability. In addition to contribute to the knowledge of the effects of the Covid-19 pandemic for families, this study further contributes to our understanding of the relationship between uncertainty and couple stability, by examining a specific context of generalized increased uncertainty and by analysing different aspects of employment and income uncertainty. The ability to distinguish between persistent unemployment, situations of employment uncertainty concomitant with the Covid-19 crisis that did not involve the loss of job (partial activity), the loss of employment occurred during the crisis, and the deterioration of income represents an important contribution to the literature on the link between uncertainty and couple separations.

## Pandemic and Economic Uncertainty in the French Context

**Fig. 1:**
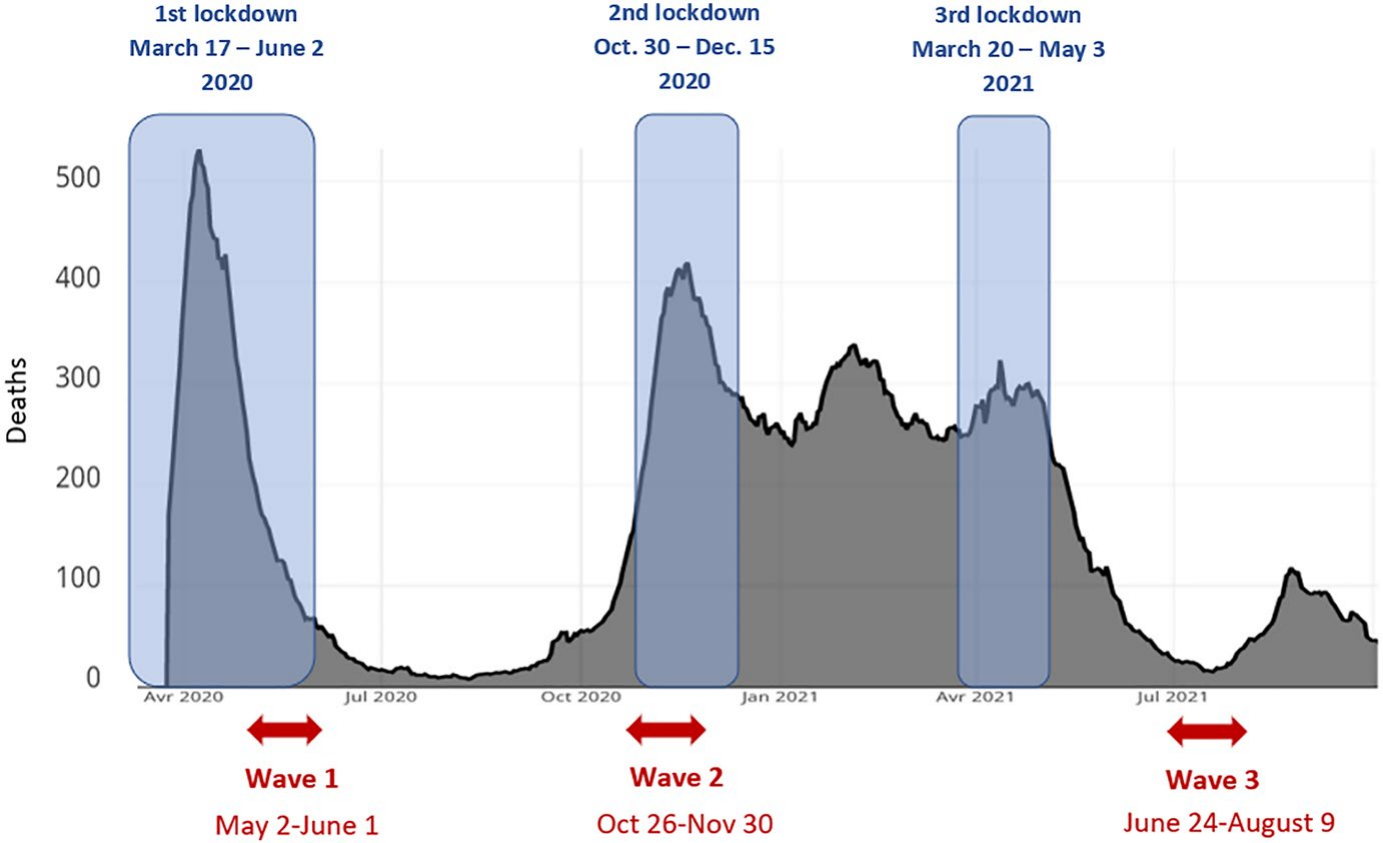
Number of hospital deaths for Covid19, lockdowns and timing of the Epicov survey. *Note*: the black line represents the evolution of daily deaths in hospitals. The arrows represent the periods of data collection of Epicov

The SARS-CoV-2 pandemic began in Europe at the start of 2020. To fight the spread of the COVID-19 pandemic, a first strict lockdown of the population was imposed from March 17 to May 11, 2020 throughout France (Fig. 1). All the so-called non-essential activities were closed and teleworking was implemented where possible. During this time, French people suffered an almost total retreat into the domestic sphere. Schools, crèches, leisure and social areas were closed. The only permitted reasons for going out were to work, to shop for basic necessities, and travel for health reasons or for family emergencies. Outings were limited to one hour within a maximum radius of one kilometre from home for individual physical activity and had to be justified by a certificate. A large number of walking areas were totally prohibited, such as parks, forests and seashores. May 11 marked the beginning of a gradual process of release from lockdown, which ended on the 2nd of June.

The second lockdown, which ran from October 30 to December 15, 2020 in mainland France, was less strict than the first. Teleworking became the rule again, when possible, but the list of essential activities was extended and many sectors were allowed to continue their activity. Crèches and schools (excluding universities) remained open. Work in factories, on farms, in construction and public works, public services could also continue. Except for work and compelling reasons, travel was once again limited to one hour within a one-kilometre radius, as in the spring.

A third lockdown took place from March 29 in some regions (from the 3rd of April in the whole of mainland France) to May 3, 2021. This lockdown was more flexible than the previous two, with traveling allowed within a ten-kilometre radius from home without time limits and extension of school holidays. While “non-essential” businesses had to close again, the list was much more limited.

**Fig. 2:**
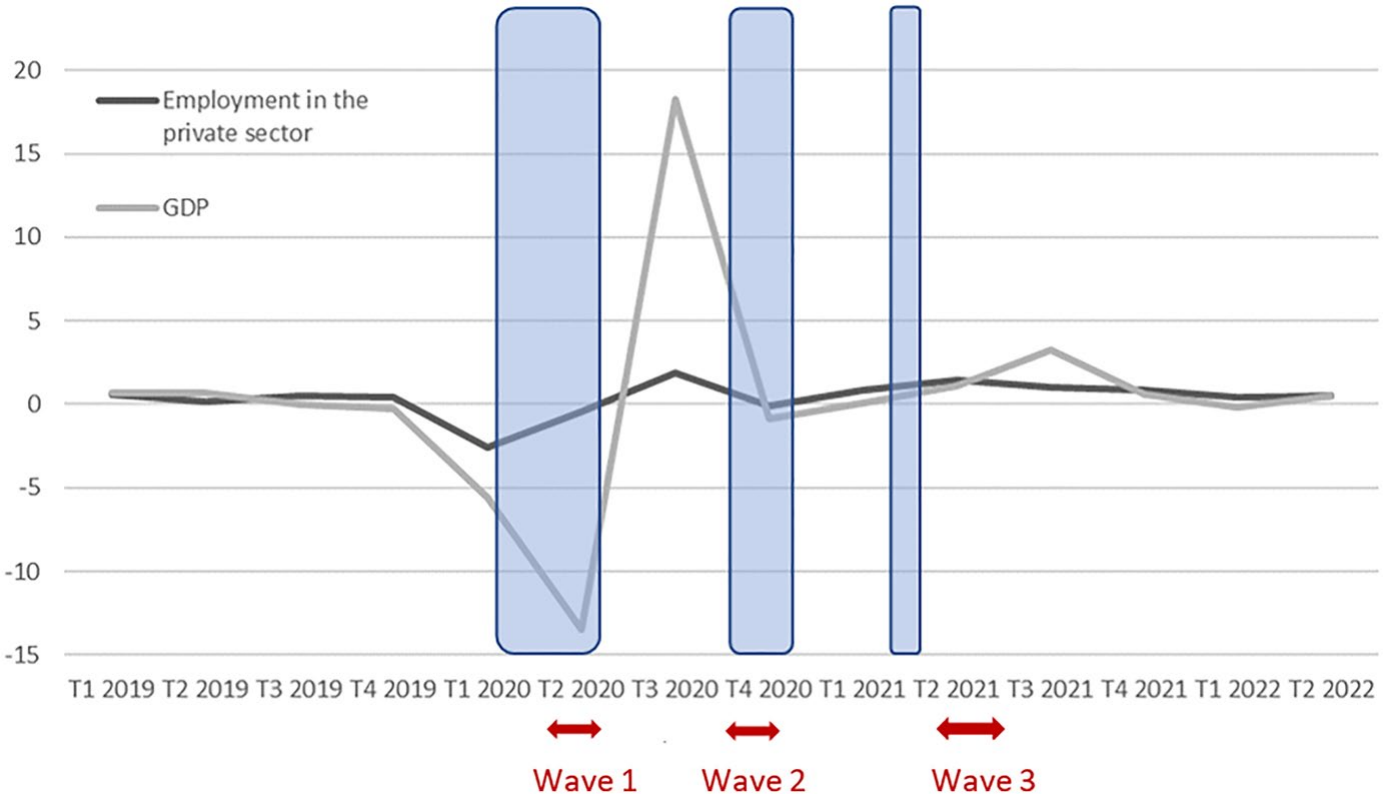
Variation of GDP and employment (%) accounts (https://www.insee.fr/fr/statistiques/2830547#tableau-) and Insee (2022). *Note*: The arrows represent the periods of data collection of Epicov. The blue surfaces the lockdowns

In the first phase of the pandemics, with the first shutdown and lockdown, France was plunging into recession: In the first quarter of 2020, gross domestic product (GDP) fell by 5.9%, and dropped by 13.8% in the second quarter of 2020 (Fig. 2). In the third quarter of 2020, economic activity had picked up. The second lockdown and the curfews put in place led again to a drop of economic activity, however with less devastating effects compared to the spring. From the first quarter 2021, economic growth turned positive again. Employment in the private sector followed these variations in economic activity, with much smaller changes^1^ (Insee, 2020). These relatively small changes in employment compared to the changes in GDP are due to protective public policies. To cope with the fall in activity and employment, since the beginning of the first lockdown the French state set up a system of partial activity to protect companies and employees whose activity was stopped or reduced. Whatever their job tenure, kind of contract (open-ended, fixed term, apprenticeship, etc.) or working time (part-time or full time), employees received a compensatory allowance of at least 70% of their previous gross pay (approximately 84% of the net hourly wage) until June 30, 2021 (60% later). While in situations of partial activity, work contracts were suspended until the company resumed normal activity. Employees were therefore guaranteed their jobs when the activity resumed. In the case of people whose fixed-term contract ended during the lock-in and whose employer did not activate the partial activity scheme, they entered the classic unemployment benefit system (the average net replacement rate on compensated days amounting to 73%, the gross replacement rate to 61%).

## Employment and Income Uncertainty and Couple Dissolution

### Background and Previous Evidence

Individual experiences of economic uncertainty, i.e. unemployment, short term employment, or deterioration of household economic conditions are risk factors for couple dissolution (Mills & Blossfeld, 2003). Furthermore, beyond the objective individual situation, future expectations, imaginaries and narratives of the future guide the decision-making process (Vignoli et al., 2020). General uncertainties about future economic conditions may be felt by all members of a society and affect even those who are not directly concerned by layoffs. It has been shown that individuals’ pessimistic perception of their own future is at least as distressing as the experience of unemployment itself (De Witte, 1999).

Competing theoretical explanations have been developed to explain the link between economic uncertainty and union dissolution. A first set of explanations predicts a positive relationship between employment and income uncertainty and the risk of divorce or union dissolution. First, according to the family stress theory, the loss of employment produces uncertainty and stress which may reduce the quality of couple relations and increase the risk of union dissolution. From an economic point of view, unemployment may be seen as an ‘unexpected’ event in the life course that reduces current financial resources (Boheim & Ermisch, 2001; Weiss & Willis, 1997), as lost earnings are only partially offset by unemployment benefits. This greater financial strain due to the decrease in the household standards of living (Greenstein, 1990; Moffitt, 2000) is a potential source of stress, which may exacerbate conflicts between partners and lead to higher risk of divorce. This increase in stress may be reinforced by the fact that unemployment brings also uncertainty about future earnings and career prospects. Hence, unemployment entails significant income uncertainties in the long term (Gangl, 2006). Moreover, losing job also leads to a loss of the social status associated with paid work and signals a lower earning potential (Cherlin, 1979), which may reduce individual’s attractiveness. Unemployment can decrease psychological well-being and self-esteem, thereby hampering relationship quality, which could in turn lead to increased marital instability (Pearlin et al., 2005; Rao, 2020). By having increased the risk of unemployment and income uncertainty, the context of the pandemic may have exacerbated the risk of couple dissolution.

A second set of explanations moderates the negative effect of economic uncertainty on union dissolution. Living in couple might help to mitigate the impact of employment uncertainty and economic hardship, and allow to better face uncertainty. As underlined by the family resilience theory (Walsh, 2003), to face a moment of crisis together can result in stronger bonds since partners offer each other social support during bad times; this solidarity during moments of crisis may reinforce the couple relationship. In economic terms, lessening financial resources increase the relative cost of separation. Situations of employment uncertainty also increase dependence on the partner’s income (Schoen et al. 2002), and decrease the ability of a couple to afford two separate housings when facing conflict, thus possibly reducing the risk of union dissolution (Cherlin et al., 2013). Last, the time availability offered by unemployment may increase participation into domestic work, which may have decreased tensions in a context of high demand for domestic work (Hupkau & Petrongolo, 2020; Kreyenfeld and Zinn 2021).

The evidence provided by micro-level research has consistently shown that individual unemployment, as well as indicators of adverse economic conditions, are associated with a higher risk of separation and divorce (Jalovaara, 2013; Hansen 2005; Doiron and Mendolia, 2012; Charles & Stephens, 2004). However, as unemployed individuals may have underlying and unobserved characteristics, such as poor physical health, or an elevated risk of depression, distress, and interpersonal tensions (Dooley et al., 1994, 1996; Norström 2014) that can hamper both job stability and couple stability (Anderson et al., 2021), it is difficult to conclude that this increased risk of separation is caused by unemployment per se. Empirical studies considering only involuntary job loss, such as displacement or plant closure, confirmed however this higher risk of divorce in case of unemployment (Charles & Stephens, 2004; Eliason, 2012).

The Covid-19 pandemic has generated massive uncertainty in all spheres of individual lives: (1) overall uncertainty, linked to the health crisis; (2) employment uncertainty, leading to a loss of status and uncertain future perspectives; and (3) income uncertainty, linked both with the deterioration of household income and the uncertain income prospects. This context provides a new opportunity to further explore the link between uncertainty and couple stability. Experiencing employment uncertainty linked with situations such as partial activity (with partial or complete reduction of working hours) during the lockdown can be considered as a more exogenous shock compared to unemployment in usual time. Hence, the Covid-19 crisis was unexpected and widespread, affecting most sectors of activity, and individuals who totally (or partially) stopped working because of it were more likely to be randomly selected than usually. Also, the selection into and stigma associated to unemployment and loss of income are less severe during a crisis or economic down-turn because they are perceived more as a collective fate and not necessarily considered as individual failure (Brand, 2015). Nevertheless, establishing a causal relationship remains delicate and difficult and goes beyond the scope of this article.

However, this context allows us to disentangle the mechanisms through which different dimensions of uncertainty can affect couple stability. Unemployment usually has three concomitant consequences: a loss of the social status and professional relationships associated with paid work, a negative income shock, and an increase in the uncertainty about future professional situation (Brand, 2015). It is thus generally difficult to disentangle the effects of these three dimensions. Instead, unemployment during the Covid-19 pandemics has been unusual in Europe because some governments, such as the French one, have guaranteed job protection, securing in a certain way future employment. Furthermore, they have provided income compensation, thus limiting income loss. Therefore, individuals who experienced Covid-related situations of employment uncertainty have been better protected both in terms of income and because most of them had the guarantee that their job would be kept, which is specific to the pandemics. Thus, this unique context provides the opportunity to separate the (temporary) loss of social status and network linked with the job from the loss of income.

### Hypotheses

In this study, we examined the risk of couple separation during the first year of Covid-19 crisis, and whether it was associated with economic uncertainty, as measured by pre-pandemic unemployment and financial conditions and the sudden change in employment and financial situation due to the economic crisis. Based on previous literature, we hypothesized that the increase in uncertainty experienced by many individuals in the Covid-19 pandemic context was linked with an increased risk of experiencing couple separation.

*HP1- employment uncertainty*: we hypothesised to observe a lower risk of separation among individuals who remained employed after the beginning of the pandemic, and increased risk when the individual experienced situations of employment uncertainty.

Previous research has shown that the type of employment uncertainty matters. For instance, layoffs lead to an increase in divorce, while there is no effect for plant closings in the US context, and this difference might be explained by the fact that the two forms of unemployment give different signals of employability and economic suitability (Charles & Stephens, 2004). Long-term unemployment also leads to higher stress levels than unemployment of short duration, since it is socially and financially more consequential (Di Nallo et al., 2022). The experience of different kinds of employment uncertainty in the pandemic context could have different consequences on individuals (and couples) wellbeing as they brought about different feelings and future prospects. We assumed that different situations would be linked with different levels of uncertainty. These are previous unemployment; Covid-related employment uncertainty such as partial activity, that allowed employed to keep their job contract but involved a reduction in working hours as well as salary, though limited by social protection; unemployment by the end of contracts during the lockdown (which might be due or not to the lockdown).

*HP2*- *differences in kinds of employment uncertainty*: We expected to observe an especially high risk of separation for those individuals who were already unemployed before the beginning of the pandemic (linked with negative selection and persistent unemployment in a moment of especially uncertain employment perspectives); to a lower extent for those who experienced the end of their contract during the first lockdown, and to even lower extent for those who experienced situations of partial activity.

Empirical findings suggest that low-income households are at a greater risk of union dissolution (Jalovaara, 2003; Hansen, 2005). Moreover, financial losses add to the psychological distress linked with the generalized health and economic crisis and contribute to stress and tensions (Cherlin, 1979). Income uncertainty and the decrease in the household standards of living may thus lead to increased risk of separation.

*HP3: change in financial conditions:* we hypothesised to observe an increased risk of separation among individuals who experienced a deterioration in financial conditions since the beginning of the first lockdown.

The individual uncertainty-couple dissolution nexus may vary according to the general context. A positive relationship between general business cycle and divorce has been observed. Divorce usually decreases when the unemployment rate (at the aggregated level) is on the rise (Gonzalez-Val & Marcén, 2017; Amato & Beattie, 2011). Due to the wide variation in economic activity as well as overall uncertainty over the year according to epidemic episodes and hardest conditions of the first lockdown, we assume that separation rates increased just after the first lockdown, with the recovery of more “normal” conditions. Moreover, in the specific case of the pandemic, unions that could not separate during the lockdown simply separated just after it was finished, through a catch-up effect.

*HP4*: *Variations throughout the first pandemic year:* we assumed that separation rates were higher shortly after the first lockdown than several months after.

The theoretical literature predicts the potential negative effects of experiencing employment uncertainty to be stronger for men than for women. Men usually earn a larger share of the household income; thus, their unemployment generates more financial strain and stress (Jalovaara, 2001). Moreover, male identity is more dependent on employment than female, meaning that men will suffer more strongly the stigma of unemployment compared to women (Gonalons-Pons & Gangl, 2021). Furthermore, women tend to be more involved in other activities, such as childrearing, that alleviate the negative consequences of unemployment (Di Nallo et al., 2021). Empirical investigations found that unemployment has stronger negative effects for men (Solaz et al., 2020), while results for female unemployment are mixed and, when significant, the size of the impact is smaller in magnitude. Indeed, gender differences in the effects of unemployment depend on the social context. Using several household panel data on couples in 29 countries, Gonalons-Pons and Gangl (2021) showed that male-breadwinner norms are a key driver of the association between men’s unemployment and the risk of separation. Thus, in societies where men are assigned the role of main provider by social norms, they tend to suffer more strongly the stigma of unemployment compared to women. On the contrary, in countries characterised by more gender equalitarian norms, men’s unemployment is not associated with higher risk of divorce than women’s unemployment (Nilsson, 2008; Charles & Stephens, 2004; Di Nallo et al., 2021).

In France, the dual-earners model is very common and women’s employment widespread, with almost 3 couples out of 4 being dual-earners^2^ (Costemalle et al., 2015). France is among the European countries where people are most supportive of gender equality ideals, behind Northern European countries (Aassve et al., 2014; Lomazzi et al., 2018). For instance, a large majority of individuals believe that men and women should share equal responsibility for family tasks and that fathers are as well-suited for looking after children, even if in practice gender inequalities persist in term of gender division of housework (Pailhé et al., 2021). We thus expect man’s and woman’s unemployment and economic situation to play quite similarly on the risk of dissolution.

*HP5: Gender differences:* Given the context of spread dual-earner families, we expected little differences depending on whether the man or the woman experienced employment uncertainty.

## Data and Methods

### Data and Sample

To answer our research questions, we used data from the French “Epidemiology and living conditions” (EPICOV) survey launched by INSERM (National Institute for Health and Medical Research) and DREES (the statistics office of the French Ministry of Health and Solidarity) to analyse the effects of the Covid-19 pandemic (Warszawski et al., 2021). A stratified random sample of 349,936 individuals aged 15 and over was drawn from the national tax register of the French statistical office (INSEE). A total of 134,391 people aged 15 or over in January 1, 2020 and living in ordinary household in mainland France, Martinique, Guadeloupe and Reunion, participated in the first wave of the survey, partially on internet and partially by telephone. A random subsample of 80% of the selected individuals were assigned to Self-computer-assisted-web interviews (CAWI), the remaining 20% to computer-assisted-telephone interviews (CATI). The first survey was conducted from May 2 to June 1, 2020, thereby covering the end of the first general lockdown period, and the first few weeks of reopening, which began on May 11 (see Fig. 1 for the Epicov survey time-line). The second wave was conducted from October 26 to November 30, 2020, thereby during the second general lockdown (107,759 respondents). A third wave was conducted from June 24 to August 9, 2021, after the third short and smooth lockdown (85,074 respondents). The rather low response rate of the first survey (37%) is in line with the strong decrease of the willingness to participate in population-based surveys worldwide in recent decades (Luiten et al., 2020). The response rate was also affected by the specific context of this survey implemented during the pandemics, by the short duration of the fieldwork, limited to four weeks, by the collection mode (mainly through CAWI), and by the over-representation of low-income individuals, who have a low response rate. Response rates at the subsequent waves were higher, at around 80% of first wave participants. As in wave 1, younger, lower educated, lower income individuals were more likely not to be followed up at subsequent waves. Non-response adjustments of all waves samples were computed using a classic two-step approach involving reweighting with homogeneous response groups and calibration.^3^ Given the large sample and the correction for non-response, the rather low response rate to the survey did not decrease the statistical power for the analysis.

The survey contains information on household composition, family status, changes in employment status and in income. Given the longitudinal design of the survey, we were able to identify couples who separated between the three waves, and examine whether this was linked with previous as well as changes in financial and employment conditions.

We selected the 63,069 individuals aged 20–75 years, who were in cohabiting with a partner (married or not) when first interviewed, and for whom information for at least the two first waves were completed. This selection of respondents interviewed at least at the two first waves may underestimate separation rate. Indeed, individual characteristics predicting non-response at waves 2 and 3 are also predictors of couple separation, and dissolution may itself increase attrition risk. However, contrary to housing-based surveys, Epicov survey followed respondents by phone, text messages and e-mails, which limits the attrition risk due to moving, regardless the move is Covid-related or separation-related. This is a strength of this survey for studying events linked to moves such as separation.

Because the first interview was conducted during the first strict lockdown, cohabiting couples were identified through household structure of the dwelling where the individual spent most of the time during the lockdown. As individuals might have moved to another dwelling for the lockdown, we only included in our sample those who declared to be living in their usual dwelling when first interviewed. This way, we avoided considering as cohabiting those couples where the respondent moved in together with their partner only temporarily to spend the first lockdown, to then return to their usual dwelling. These couples would be counted as a separating if we did not exclude individuals who declared they were not living in their usual dwelling during the lockdown. Nevertheless, as information about the duration of the union is unknown or partial, we could not exclude people whose partner moved in for the lockdown. Thus, our sample is likely to include some couples who were very recently formed, some of whom might even have not being cohabiting if it was not for the lockdown, thus increasing the amount of “frail unions” observed compared to what we would have observed using other data sources. As a robustness check, we excluded individuals who were not in union two years before the survey, information included in the sampling frame of the tax register. Our selection criteria also exclude couples who decided to spend the lockdown elsewhere than their usual housing, in a second house. An alternative specification includes them and results are not sensitive to their inclusion.

### Separation

We identified separations using information about household composition at waves 1 to 3. Therefore, individuals who declared that they were cohabiting with a partner at wave 1 and were not cohabiting with a partner at wave 2 were considered as separating between wave 1 and 2. This means that our first variable on separation considers a period of time of around 6 months (176 days on average) between May/June and November 2020, so just after the first lockdown. Individuals who were cohabiting at wave 2 and not cohabiting anymore at wave 3 were considered as separated between the two waves, thus between November 2020 and July 2021 (around 8 months, 242 days in average). Overall, in our sample of coresident couples in wave 1, 2,514 individuals separated between wave 1 and 2 (separation V1–V2); 550 individuals separated between wave 2 and 3 (separation V2–V3). It is important to highlight that the two samples exposed to separation in the two time-periods differ as individuals who are cohabiting at wave 2 are those who survived the first lockdown and will thus be already selected compared to those in couple at wave 1.

### Employment and Income Uncertainty

We operationalized uncertainty through measures of initial conditions (before the first lockdown) and change in employment and financial situation due to the pandemic crisis and the lockdowns.

Initial conditions were measured by the employment situation before the first lockdown (individuals who were employed, unemployed, student or inactive before the beginning of the lockdown) and household income (whether the household belonged to the first tercile of income in the calendar year preceding the lockdown according to fiscal data).

We considered three additional measures of change in employment uncertainty, measured at both wave 1 (thus indicating a change occurred during the 1st lockdown) and wave 2 (thus indicating a change occurred in the months after the 1st lockdown):

(1) Whether the individuals experienced an interruption of work because their activity was not included among the ‘essential activities’ and could not be carried out remotely (“activité partielle” or “chômage technique” which we call *partial activity*. These individuals were covered by the compensatory allowance and the maintaining of the employment relationship.

(2) Whether the working contract of the individuals ended, which we call *end of contract*. In this case, individuals received an unemployment allowance if their employment record allowed it and there was no employment guarantee.

(3) Whether the individual was not employed before the beginning of the 1^st^ lockdown, and started working during or in the months following the 1st lockdown.

Because uncertainty about future professional situation could exist even when job is protected, particularly in uncertain times such as the Covid period, it is important to take into account the feelings about financial situation, beyond income and job situations. In the second wave only, a question about feelings of employment uncertainty was asked. Thus, we were able to compare the association between objective and subjective measures of employment uncertainty and the risk of separation between the second and the third wave.

As concerns income uncertainty, we considered a measure of whether the individual declared to have experienced a change in the household financial situation since the beginning of the pandemic. We focussed on individuals who experienced a worse situation, operationalised as a dummy variable equal to 1 whether the individual declared their financial situation had deteriorated, and 0 otherwise. The question was asked at both wave 1 and wave 2. We also included deciles of pre-pandemic household income.

### Statistical Analyses

First of all, we described trends in separations in our sample, by individual main characteristics (age, marital status, number of children, age of the youngest child)^4^ and professional situation (employment status, type of employment and type of contract) before the first lockdown. In the Appendix (Figures 8 and 9), we show the determinants of experiencing employment and income uncertainty in the two considered sub-periods with multivariate logistic regression models.

To examine whether employment and income uncertainty was linked with an increased risk of experiencing separation, we implemented logistic models on the risk of couple separation. We run models (1) for the risk of separation between wave 1 and 2, thus examining the role of employment and income initial conditions and of changes due to the first lockdown; (2) for separation between wave 2 and 3, considering both the effect of initial conditions, change in employment during the first lockdown, and some months after to examine the persistence in time of the effects of uncertainty.

We chose to run joint models including both employment and income uncertainty, to explore whether one of the two dimensions of uncertainty prevailed over the other in predicting couple separation.^5^ All models controlled for respondent’s age, gender, education, and marital status, as well as household number of children, size of the dwelling (whether less than one room for person living in it) before the first lockdown.

## Results

### The First Months of the Pandemic Were Particularly Challenging for Couples

Table 1 shows that, among the 63,069 individuals aged more than 20 up to 75 years who were cohabiting at the first wave and followed up to the second wave, 4.2% separated within around 6 months starting at the end of the first lockdown (between May–June and October–November 2020, when the two first waves were collected). Among the 49,277 individuals who were cohabiting at the second wave and were followed up to the third wave, 1.35% separated in the following 8 months (from end October-end November 2020 to end June-early August 2021, when the third wave was collected). Our data thus suggest high rates of separation observed within the first semester, while rates in the second semester seem to be more in line with what generally and previously observed, in line with HP4. The large gap in separation rates between the two periods may be due to an accelerating process of the lockdown, that affected the most fragile couples who could not withstand the crisis. It could also be due to catching up on separations that could not take place during lockdown. If we consider the extreme scenario with no possibility of separation during the first lockdown, the rate of separation of 4.2% corresponds to a period of nearly 8 months (instead of 6); this rate is still three times higher than what is observed in the following 8 months.

**Table 1:** Number and rates of separation, by age group

	< 30 years	30–40 years	40–50 years	50–60 years	> 60 years	Total
*On Epicov sample*						
Separation V1-V2 (weighted rate)	467	478	505	514	550	2,514
	(10.38%)	(4.53%)	(3.29%)	(3.48%)	(3.40%)	(4.20%)
N	4,040	11,857	14,594	14,384	18,194	63,069
Separation V2-V3 (weighted rate)	92	143	141	100	74	550
	(4.51%)	(2.61%)	(1.15%)	(0.52%)	(0.21%)	(1.35%)
N	2,566	8,695	11,381	11,560	15,075	49,277
Separation V1-V3 (weighted rate)	559	621	646	614	624	3,064
	(13.72%)	(8.28%)	(5.52%)	(5.25%)	(2.90%)	(6.08%)
*On couples in Epicov survey as defined in fiscal data *						
Separation V1-V3 (weighted rate)	51^2^	300	386	328	343	1416
	(3.86%)	(7.18%)	(4.40%)	(3.01%)	(2.27%)	(4.06%)
Yearly-corrected separation rate A^1^	3.31%	6.15%	3.77%	2.58%	1.95%	3.48%
Yearly-corrected separation rate B	2.90%	5.39%	3.30%	2.26%	1.70%	3.05%
N	1,186	7,516	10,612	10,692	14,384	44,231
*On fiscal administrative data one year before Covid*						
Separation 2018	53,494	80,318	90,437	63,912	39,357	327,518
	(6.64%)	(4.45%)	(3.41%)	(2.41%)	(1.01%)	(2.78%)
N	805,202	1,803,406	2,652,764	2,653,755	3,882,610	11,797,737

^1^A: corrected for duration of 12 months B: corrected with duration including the period of first lockdown

^2^For young people, the definition of couple according to fiscal data differs substantially from the one according to the Epicov survey, since younger individuals only rarely are tax filers or the partner of a tax filer. For this reason, the rates showed here are calculated on individuals 25 years or older

We tried to compare these separation rates with those observed in usual times. Finding a comparable representative data source to observe dissolution risk including both married and unmarried couples is not straightforward.^6^ We used the last administrative exhaustive database from fiscal data 2019 that provides information on changes in household composition between January 2018 and January 2019. It has the advantage to observe and follow households even in case of move. By comparing the family situation between two calendar years, we were able to estimate a yearly separation rate in fiscal data; the observation period of being at risk of separating is thus about 1 year in both cases. For comparability issue, we restricted the sample to the same definitions of couples in both data sources.^7^ We selected in Epicov the individuals in couple as defined by fiscal data (individuals in couple with or without children, tax filer respondent either fiscal referent, or legal or de facto partner of the fiscal referent) or in other words, individuals in couple interviewed by the survey that would also be identified as in couple in the fiscal data. This eliminates recently formed couple (since less than one year) and some young people under 25 who are not yet tax filers, that is why we restricted the youngest age category to 25–30 years. For the other age groups, we could compare the breakup rates along one year or roughly (for Epicov survey). The results of this comparison are shown in the last two rows of Table 1. Overall, the observed weighted rate in 2020 for individuals older than 25 (4.06%) was substantially higher than the approximately comparable annual rate observed in France in 2018 (2.78%). To make the yearly rates comparable when we considered the risk of dissolution from the beginning of the first lockdown, we corrected for the longer period of observation (A: rate*12/14) during 2020 than 2018 and for the extreme scenario (B: rate*12/16). The corrected yearly rate (3.48%) remains higher than the comparable annual rate observed in France in 2018, even in the extreme scenario (3.05%).

Looking at separations by age, we can see that, whatever the age, separation rates were systematically higher during the first year of Covid-19 pandemic compared to 2018 in the fiscal data on similar population, except for young people aged 25–29 years old. These results suggest that many couples might have experienced difficulties facing the challenges related to the first months of pandemic and thus decided to separate. This is particularly true for prime-age individuals (between 30 and 40 years) that were by far the most likely to separate compared to 2018.

### Who Separated?

**Fig. 3:**
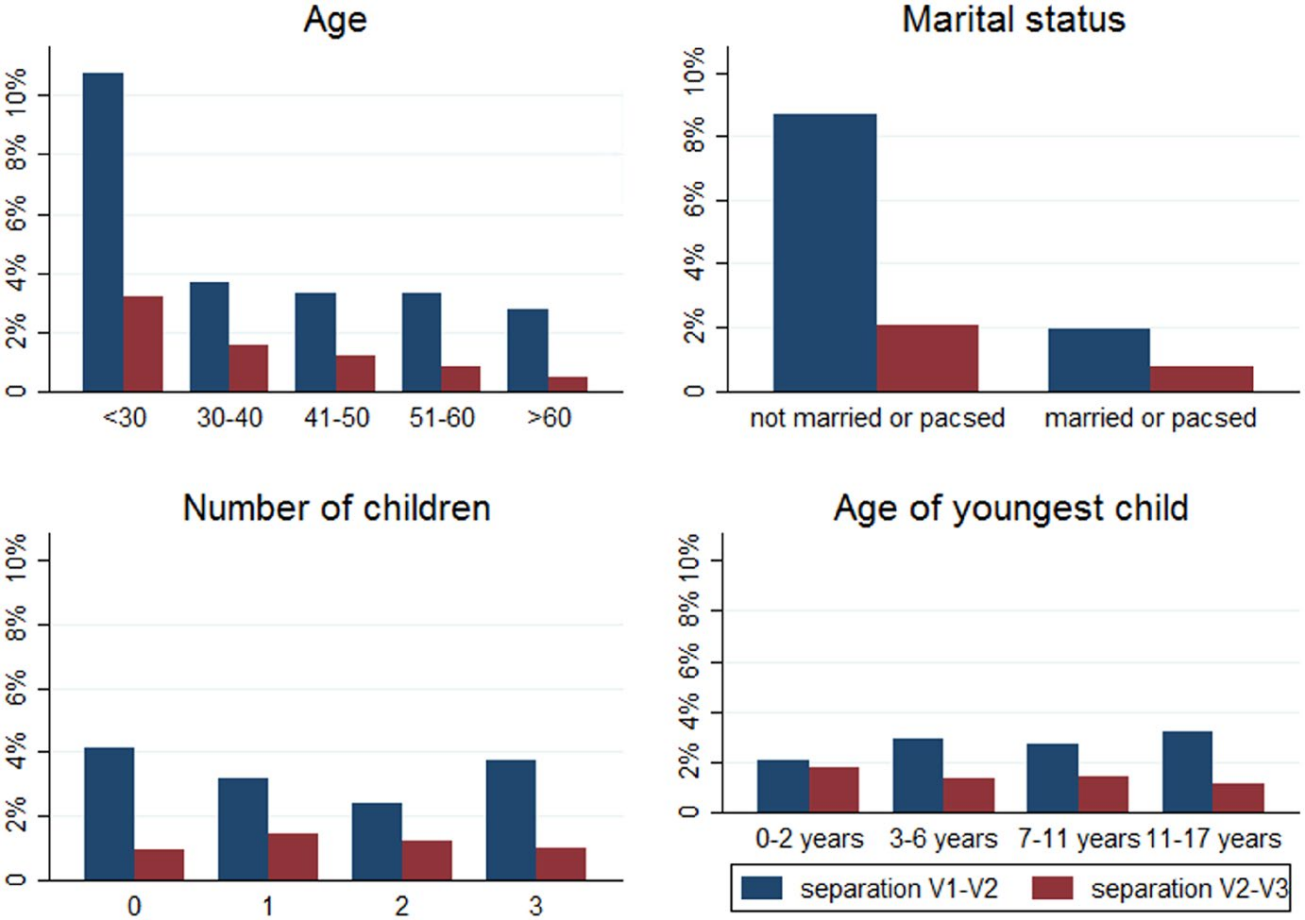
Trends in separation rates by main individual characteristics

**Fig. 4:**
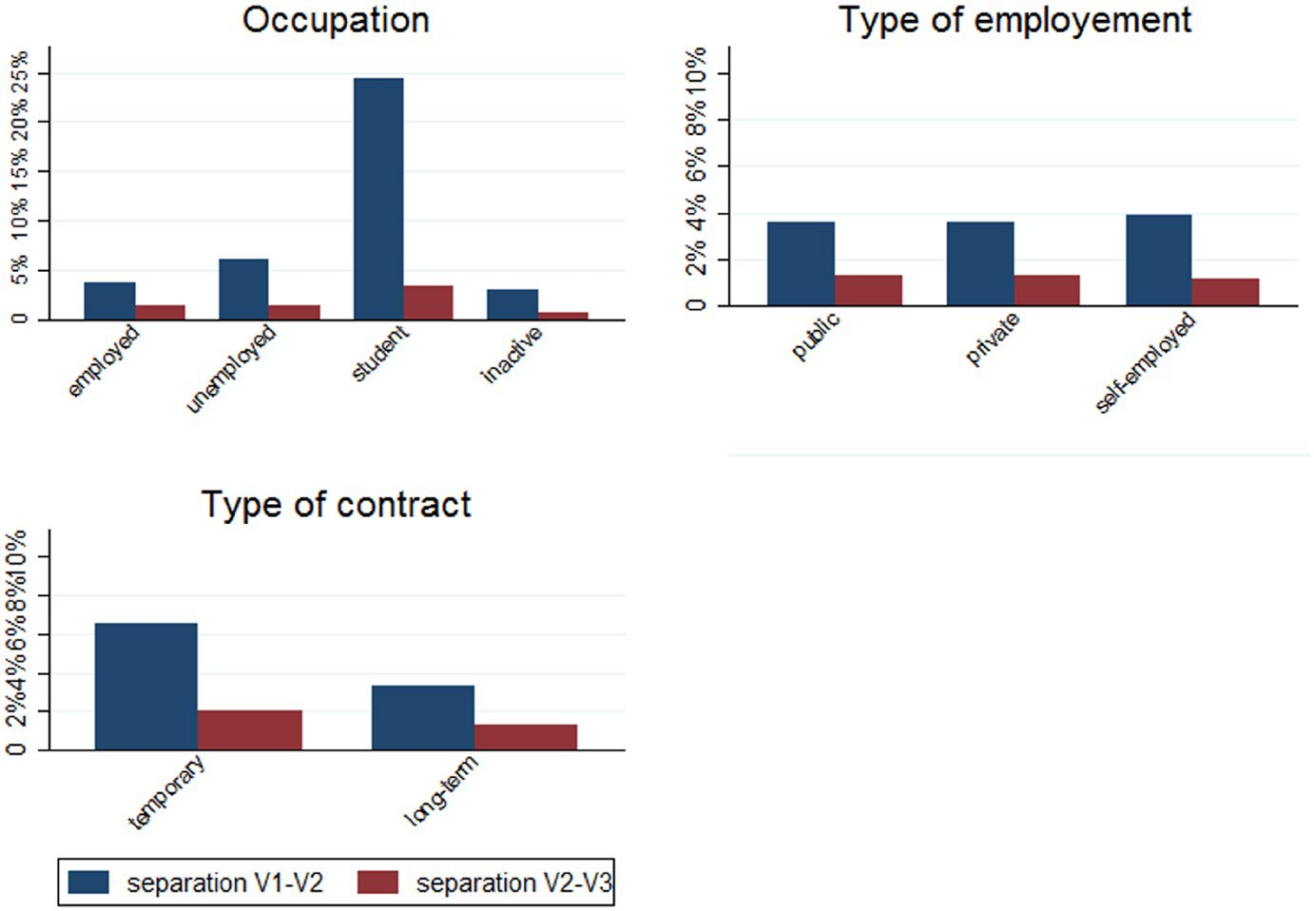
Trends in separation rates by employment situation before the first lockdown

Looking at differences in separation rates depending on individuals’ main demographic characteristics (Fig. 3), Epicov data show that married couples or those who are in a civil union were less likely to separate compared to other couples, especially in the months following the first lockdown (first period); childless couples were more likely to separate compared to couples with one or two children, however the rates increased among couples with three children or more; individuals with toddlers at home were less likely to separate in the months following the first lockdown, but slightly more likely to separate later. As concerns separations rates across individuals with different occupation before the first lockdown (Fig. 4), the high rate of separation observed among the younger population is also reflected by the high rates among students, especially in the first period. In the second period, separation rates were not substantially different depending on the occupational status, which might be partly explained by the fact that couples who were in a more difficult situation before the pandemic might have already separated in the months following the first lockdown. On the other hand, couples of individuals in rather unstable situations, such as students or where one of the two partners was unemployed who managed not to separate during or just after the first lockdown might have been the most stable ones and thus less likely to separate. Among employed individuals, those who had a temporary contract were more likely to separate compared to those with a long-term one, while rates were not substantially different depending on whether the individual was employed in the private or public sector, or self-employed.

### The Role of Economic Conditions and Uncertainty

Levels of employment and income uncertainty substantially increased during the first year of Covid-19 pandemic (Table 2). More than 20% of the sample at both waves declared that their financial situation had deteriorated since the beginning of the pandemic (around 26% in the first and just over 20% in the second wave), and the proportion was higher among individuals who later separated. 19% of individuals experienced partial activity during the lockdown, again with a higher proportion observed among individuals who separated. The proportion of individuals who lost their job was of course substantially lower (around 1% of the sample at both waves), but the proportion was almost the double for individuals who later separated.^8^ This suggests that exploring the relation between uncertainty and separation might be crucial. Looking at the proportion of individuals who experienced changes in employment and income uncertainty (Table 3), we can also see that the two dimensions are strictly linked.^9^ In particular, more than half of the individuals who experienced job loss and more than 40% of those who experienced partial activity during the first lockdown declared that their financial condition had worsened. The proportions were even higher for individuals who experienced employment uncertainty afterwards. However, there was an important proportion of individuals who, despite experiencing employment uncertainty, did not experience income uncertainty as measured by a deterioration in financial conditions. Moreover, a significant share of individuals who were continuously employed declared a deterioration of financial situation. This confirms that it is interesting to examine how both uncertainty dimensions might be separately linked with the risk of separation.

**Table 2:** Descriptive characteristics of sample, by separation between V1 and V2 and between V2 and V3

Variable (%)	No separation	Separation V1-V2	Separation V2-V3	Tot
Age (years)	51.1	45.8	43.2	50.8
Childless	59.1	66.1	51.8	59.3
One child	16.2	13.9	21.3	16.2
Two children	18.5	12.6	20.7	18.3
Three or more children	5.9	6.7	5.4	5.9
Married/in civil union	77.1	41.2	52.6	75.5
Household Income D1	4.4	8.6	6.3	4.5
Household Income D2-D3	7.8	14.1	10.5	8.0
Household Income D4-D5	11.5	13.9	15.4	11.6
Household Income D6-D7	17.7	16.1	18.0	17.6
Household Income D8-D9	23.8	20.9	29.3	23.8
Houdehold Income D10	13.4	10.2	10.0	13.3
Primary education	10.5	11.4	6.9	10.5
Professional low	20.9	20.5	16.3	20.8
Secondary	18.4	19.9	18.9	18.5
Tertiary low	16.5	15.1	19.4	16.5
Tertiary high	33.4	32.6	38.3	33.5
Employed before lockdown	62.2	63.0	75.1	62.3
Long-term contract	92.1	84.5	87.3	91.8
Unemployed	3.6	6.3	4.9	3.7
Student	0.4	3.8	1.8	0.6
Inactive	33.7	26.8	18.1	27.9
Small dwelling	6.8	14.2	12.2	7.1
End of contract during 1st lockdown	1.0	1.8	2.2	1.0
End of contract after 1st lockdown	1.2	2.7	2.2	1.2
Partial activity during 1st lockdown	19.1	21.4	22.3	19.2
Partial activty after 1st lockdown V2	3.0	–	4.3	3.0
Started work during 1st lockdown	1.8	–	1.8	1.9
Started working after 1st lockdown	15.6	–	18.5	15.7
Feeling of employment uncertainty at V2	17.0	–	24.2	17.2
Worse financial condition at V1	22.3	29.5	31.4	24.1
Worse financial condition at V2	18.7		27.1	20.6
N	48,727	2,514	550	51,791

All variables except for change in employment and financial conditions refer to wave 1.

**Table 3:** Relation between dimensions of uncertainty, weighted proportions

	Worse financial situation since 1st lockdown- V1 (%)	Worse financial situation since 1st lockdown- V2 (%)
Continuously employed	31.2	23.3
Unemployed before 1st lockdown	39.3	30.5
Student before 1st lockdown	19.6	25.6
Inactive before 1st lockdown	14.7	13.6
End of contract in 1st lockdown	54.4	46.1
Partial activity in 1st lockdown	43.6	31.2
Started working in 1st lockdown	35.9	30.4
End of contract after 1st lockdown	*–*	64.7
Partial activity after 1st lockdown	*–*	55.0
Started working after 1st lockdown	*–*	25.3
Total	24.3	20.6

**Fig. 5:**
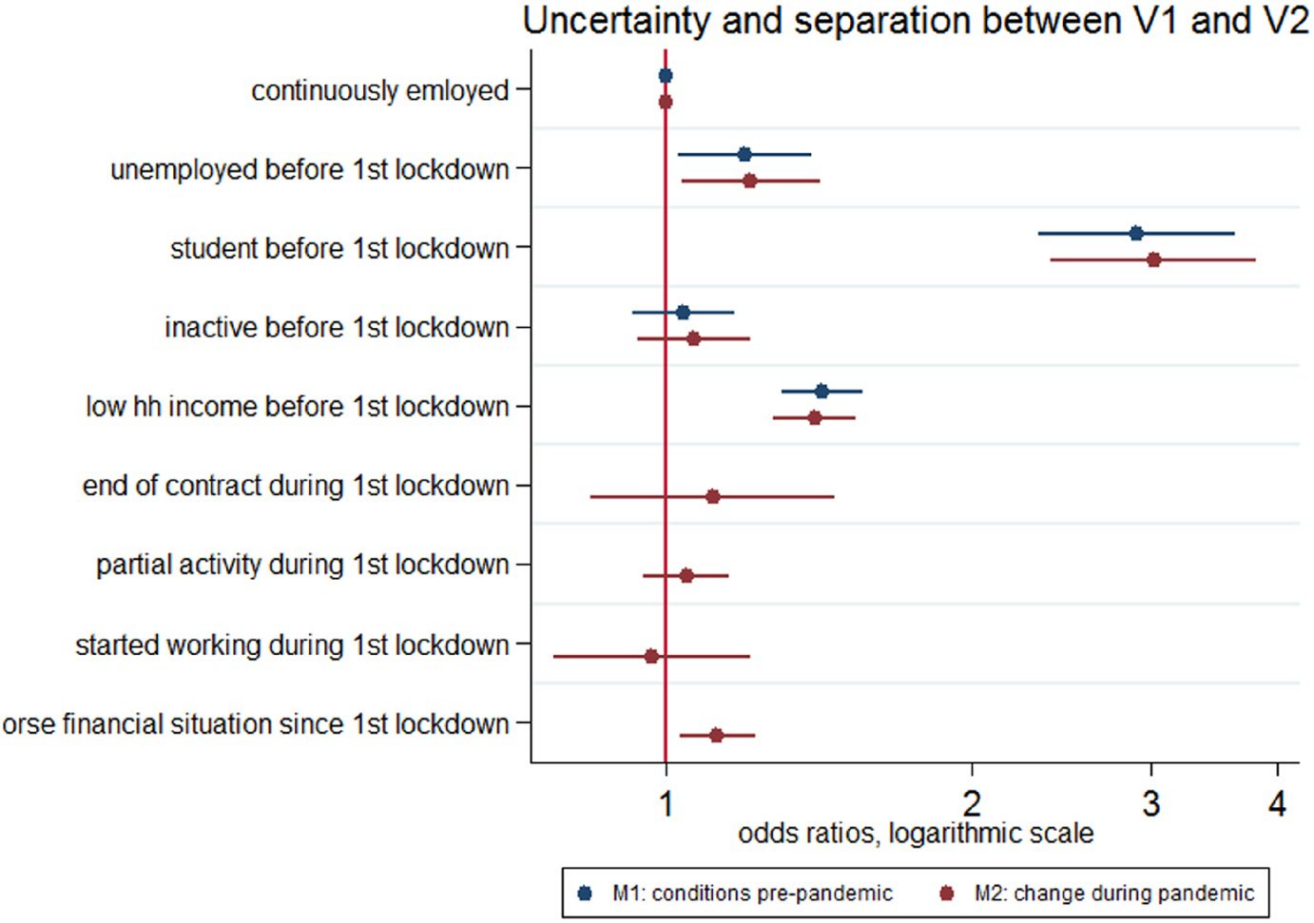
Uncertainty and separations between V1 and V2, results from joint models (1) and (2) *Note*: results from logistic models; all models control for age, gender, marital status, number of children, education, missing income, size of the dwelling. Full results can be found in Table 3, columns 1 and 2

Initial employment and economic conditions (before the first lockdown) were important drivers of couple stability in the 6 months following the end of the first lockdown (Fig. 5). Compared to continuously employed individuals, students and to a less extent unemployed ones had a significantly higher risk of dissolution. Also, those belonging to poorest households were more likely to break-up. Once these initial conditions were taken into account, differently from what hypothesized (HP1), situations of employment uncertainty during the first lockdown were not associated with a significantly increased risk of couple separation. Neither the loss of employment by partial activity, nor the loss of employment by end of contract was associated with a significantly increased risk of separation compared to stable employment (though the risk ratio for loss of the end of contract is higher than one). Thus, in line with our second hypothesis (HP2), longer-term unemployment (individuals who were unemployed already before the beginning of the lockdown) was more detrimental for couple stability than recent unemployment during the lockdown (all coefficients resulting from the models are shown in Table 4). The dissolution risk during lockdown might have been not sensitive to unemployment because both the negative selection was less severe and the duration of the unemployment spell shorter.

**Table 4:** Employment and income uncertainty and dissolutions between V1 and V2 and between V2 and V3

	(1)Joint model	(2)Joint model	(3)Joint model	(4)Joint model	(5)Joint model
	Pre-pandemic conditions	Change during 1st lockdown	Pre-pandemic conditions	Change during and after 1st lockdown	Feeling of employment uncertainty
	Separation V1-V2	Separation V1-V2	Separation V2-V3	Separation V2-V3	Separation V2-V3
Female	1.000	0.983	0.985	0.980	0.995
	(0.10)	(0.39)	(0.17)	(0.23)	(0.06)
*Age (ref.* < *30)*					
30–40 years	0.731**	0.712**	0.691**	0.691*	0.687*
	(4.37)	(4.24)	(2.43)	(2.42)	(2.36)
40–50 years	0.851*	0.863**	0.590*	0.590**	0.618**
	(2.32)	(1.80)	(3.30)	(3.29)	(2.90)
50–60 years	0.823**	0.817**	0.408**	0.413**	0.412**
	(2.88)	(1.47)	(5.30)	(5.22)	(5.24)
60–75 years	0.740**	0.747**	0.310**	0.322**	0.357**
	(3.45)	(2.78)	(5.10)	(4.90)	(4.33)
Married or in civil union	0.251**	0.254**	0.515**	0.521**	0.516**
	(28.27)	(26.95)	(6.52)	(6.40)	(6.50)
Number of children (ref: childless)					
One	0.610**	0.607**	0.930	0.923	0.896
	(7.41)	(7.18)	(0.58)	(0.65)	(0.85)
Two	0.553**	0.542**	0.789	0.786	0.786
	(7.97)	(7.86)	(1.72)	(1.75)	(1.64)
Three or more	0.737**	0.710**	0.595*	0.591*	0.551*
	(3.08)	(3.30)	(2.38)	(2.41)	(2.61)
*Education (ref: lower)*					
Professional low	0.962	0.959	1.059	1.059	1.101
	(0.50)	(0.53)	(0.31)	(0.29)	(0.47)
Secondary	0.930	0.904	1.078	1.072	1.069
	(0.91)	(1.22)	(0.38)	(0.35)	(0.34)
Tertiary low	0.954	0.936	1.223	1.218	1.201
	(10.62)	(0.84)	(1.09)	(1.06)	(0.99)
Tertiary high	0.934	0.907	1.068	1.066	1.035
	(0.82)	(1.11)	(0.33)	(0.32)	(0.17)
Small dwelling	1.697**	1.717**	1.583**	1.551**	1.618**
	(7.68)	(7.51)	(3.20)	(3.05)	(3.27)
*Occupation before 1st lockdown (ref: employed)*					
Unemployed	1.117*	1.136*	1.010	0.950	0.970
	(1.83)	(1.32)	(0.05)	(0.24)	(0.15)
Student	2.881**	2.939**	1.497	1.502	1.540
	(7.91)	(7.64)	(1.14)	(1.16)	(1.22)
Inactive	1.007	1.014	0.814	0.815	0.767
	(0.10)	(0.18)	(1.25)	(1.23)	(1.54)
Low income before ld	1.405**	1.427**	1.080	1.042	1.076
	(6.09)	(6.37)	(0.62)	(0.33)	(0.57)
Missing income	1.130	1.144	0.855	0.857	0.818
	(1.65)	(1.77)	(0.87)	(0.86)	(1.08)
*Change in occupation*					
End of work in 1st ld		1.137		0.851	0.912
		(0.77)		(0.36)	(0.21)
Partial activity in 1st ld		1.029		0.502	0.478
		(0.47)		(1.57)	(1.66)
Started working in 1st ld		1.087		0.904	0.902
		(0.65)		(0.33)	(0.32)
End of work after 1st ld				1.379	1.409
				(0.89)	(0.95)
Partial activity after 1st ld				1.965	1.841
				(1.39)	(1.22)
Starts working after 1st ld				1.682	1.599
				(1.18)	(1.05)
Feeling of employment uncertainty at V2				1.234*	1.27*
				(1.89)	(2.01)
*Change in financial situation*					
Worse financial situation at V1		1.096*		1.135	1.103
		(2.28)		(1.18)	(0.87)
Worse financial situation at V2				1.234*	1.191*
				(1.89)	(1.49)
*N*	62,904	50,207	49,175	49,136	46,167

^*^p < 0.1; **p < 0.05

The associations between deterioration in financial situation and risk of separation went in the expected direction, with increased risk of separation observed among individuals who declared their financial situation to be worse since the beginning of the pandemic, net of the actual income level observed before the beginning of the pandemic (HP3).

**Fig. 6:**
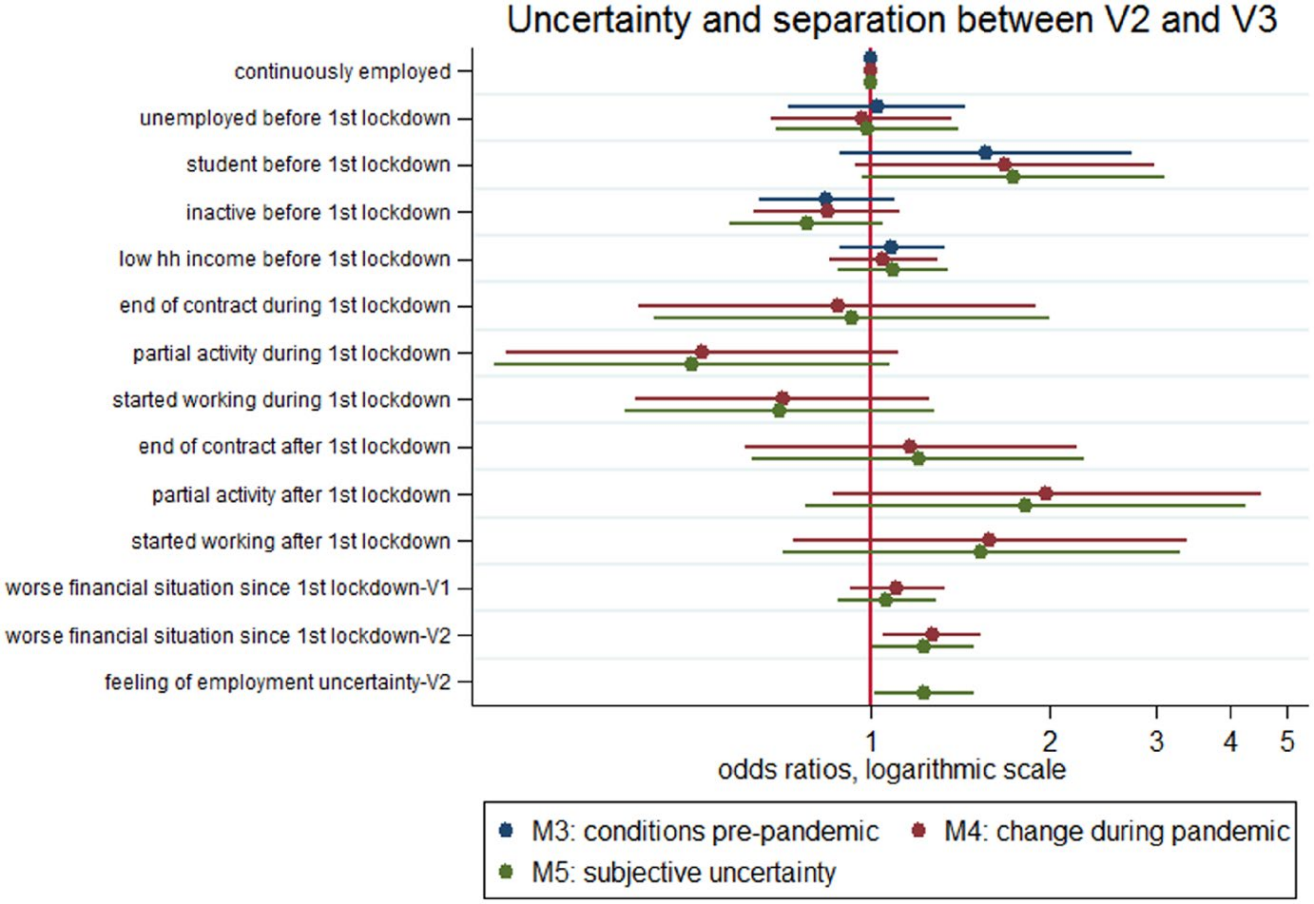
Uncertainty and separations between V2 and V3, results from joint models (3), (4) and (5) Note: results from logistic models; all models control for age, gender, marital status, number of children, education, missing income, size of the dwelling. Full results can be found in Table 4, columns 3 to 5

Among those still in union in Autumn 2020, the experience of employment uncertainty after the beginning of the first lockdown (in any of the two time-periods) was not significantly associated with the risk of separation between V2 and V3. To experience partial activity during the first lockdown in Spring 2020 seemed to have no or rather protecting effects on the risk of separating within the subsequent 8 months (V2–V3 between November 2020 and July 2021) compared to when it occurred later (Table 3, column 4 and Fig. 6), however the two risk-ratios are not significantly different from one. Moreover, the risk of separation was not correlated with experiencing the end of contract in any of the two time-periods. However, it is important to consider that the number of separations occurring in the second semester was quite small (550 in total). Our non-significant results might thus be at least partly linked to a lack of statistical power. In addition, as already mentioned, the couples who were cohabiting at V2 and thus exposed to the risk of separation in the second semester were a highly selected proportion of those cohabiting at V2 (who had “survived” the first lockdown together).

On the other hand, subjective feelings of employment uncertainty declared at wave 2 were significantly related with a higher risk of separation later. Also, those who still faced in Autumn 2020 a deterioration in financial conditions since the first lockdown had a significantly higher risk of separation during the second period of observation.

In sum, it seems that financial deterioration (and income uncertainty) has more permanent effect on couple dissolution over the periods than employment situation in a context of highly protected unemployment.

### Heterogeneity by Gender

Since we did not have information on both partners’ employment and financial conditions, we proxied the estimation of gender differences in the effect of employment and income insecurity by stratifying the sample by gender. Because of sample size issues, we could explore gender differences only for separations occurring just after the first lockdown (between V1 and V2).

**Fig. 7:**
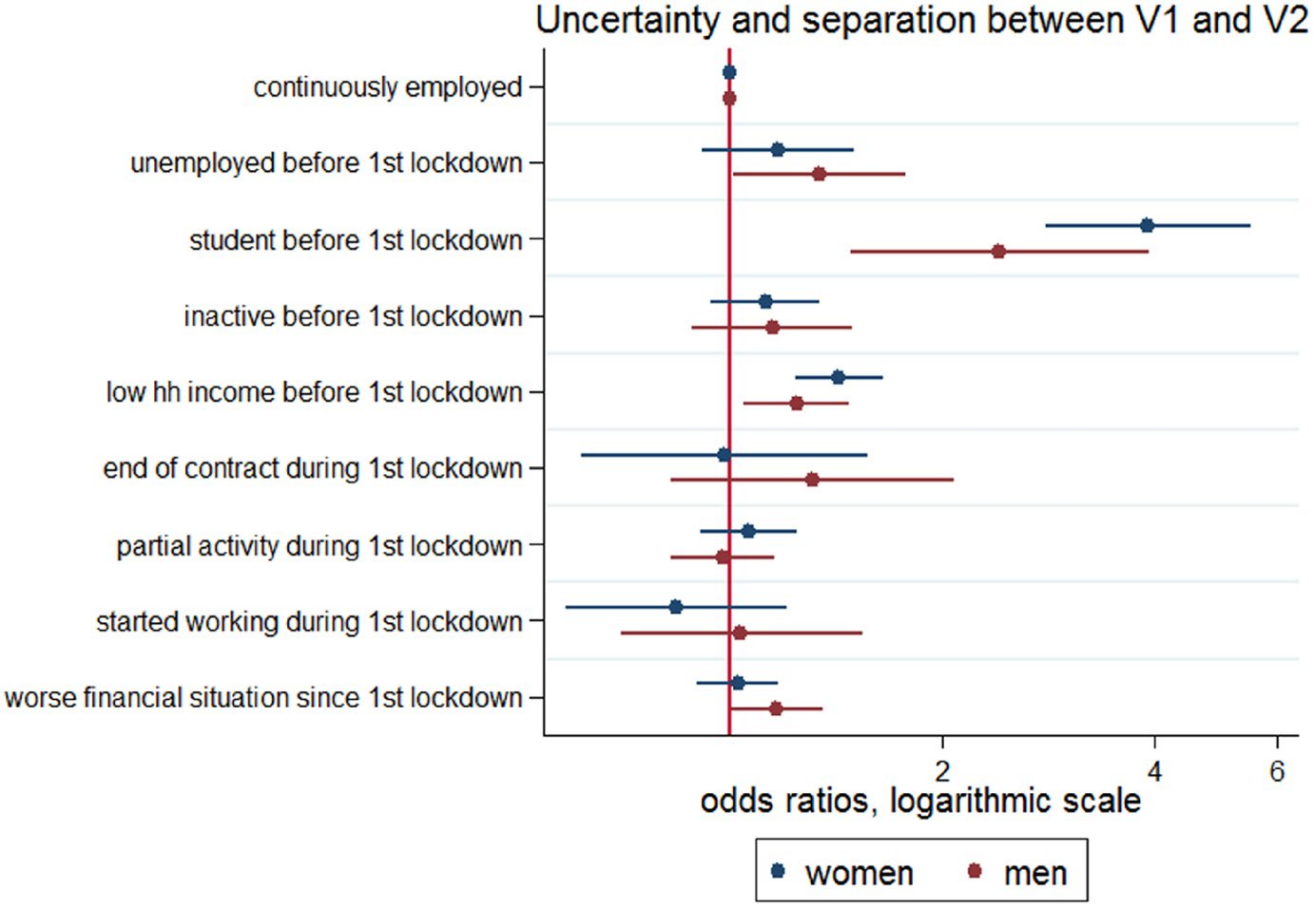
Uncertainty and separations in V1-V2, by gender *Note*: results from logistic models; all models control for age, gender, marital status, number of children, education, missing income, size of the dwelling

**Fig. 8:**
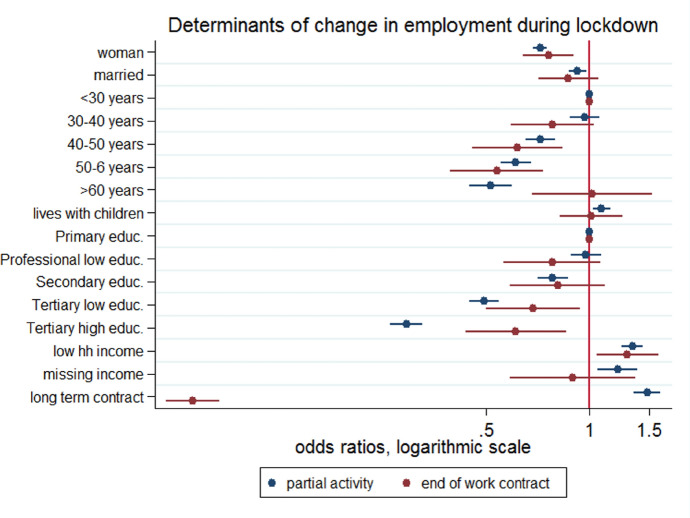
Determinants of experiencing employment uncertainty

As concerns initial economic conditions, we can observe that male as female students were more likely to separate (Fig. 7); both male and female unemployed also had a higher risk of dissolution in comparison with employed individuals (but not significant for women). The risk seemed higher for men. Lower income before the pandemic was positively associated with a higher risk for both women and men. We did not expect to find gender differences for income since the definition we used here is based on the household income and income tax data (not self-declared).

**Fig. 9:**
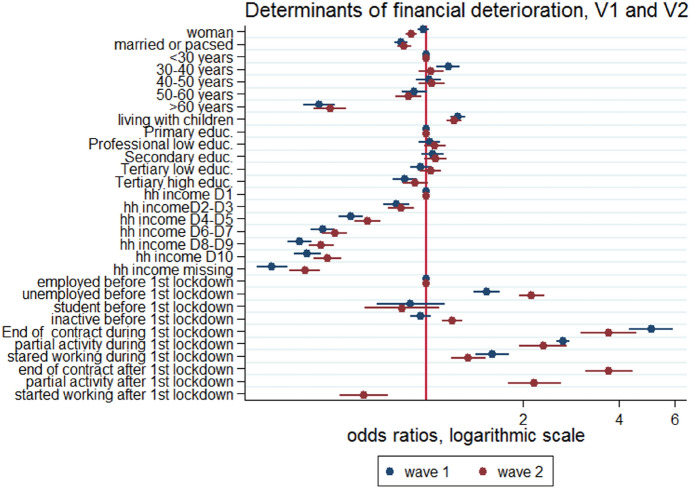
Determinants of experiencing income uncertainty

As concerns changes due to the lockdown, we observed a positive and significant association between a deterioration in the financial situation declared by men and the risk of separation. On the contrary, the association was not significant for women. This suggests that, while both partners were supposed to describe the same change in household financial situation, the effects seem to be gendered. Figure 9 in appendix shows that men are slightly more likely to declare a deterioration of the household financial situation. One explanation may be that men are more aware, and/or worried about the deterioration of the household financial situation than women because they are usually in charge of household financial management and they earn in average more. Because of their position of main breadwinner, they may feel more responsible of household financial situation. Therefore, they may be more stressed, which might play a stronger role on couple stability.

As for the overall model, we did not find any significant effect for newly unemployed either among men or among women, confirming that unemployment during the Covid pandemic had less effects than long-term unemployment, likely because of its shorter and less stigmatising nature.

**Fig. 10:**
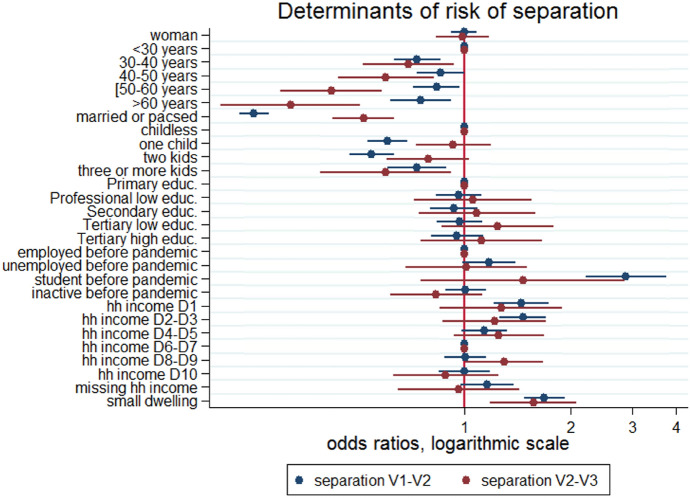
Determinants of separation

The coefficients of the control variables (shown in Figure 10 in appendix) tend to confirm what we observed in the descriptive analyses: separations occurring in the first semester were substantially and significantly more common among unmarried couples, young and childless individuals, while no significant educational gradient could be observed. Coefficients are usually similar in size, but less statistically significant for separations occurring in the second semester. As mentioned, this might partly be due to a lack of statistical power linked to the smaller number of separations occurring.

## Conclusions and Discussion

By examining couple separation rates and their association with different measures of employment and income uncertainty during the pandemic crisis, this analysis adds to the scarce literature that addresses the role of the Covid-19 pandemic on family structures. Using longitudinal data from a large representative sample followed during the first year of the pandemic crisis in France and comparing with fiscal data, we showed that the proportion of separations was higher than in normal times. Separation rates were particularly high in the 6 months following the first strict lockdown, between early June and mid-December 2020, and were substantially lower in the following 8 months. This suggests an important effect of the first strict lockdown on the quality of couple relationships, when couples lived in quasi-isolation, but a return to a more normal situation afterwards. Many couples, of all ages, but especially in their thirties, might have experienced difficulties facing the challenges related to the first months of Covid-19 pandemic and thus decided to separate.

Many people experienced increased economic uncertainty during the pandemic; situations of partial activity were specific to the first lockdown, but the deterioration of financial conditions was more persistent. In line with what hypothesized, our results show that, overall, income and to some extent employment uncertainty experienced during the Covid-19 pandemic were significantly associated with a higher risk of separating, after controlling for individual characteristics such as education, employment and income level before the beginning of the pandemic. These results suggest that, although mechanisms increasing couples’ resilience during the Covid-19 pandemic might have been in place, the negative consequence of uncertainty seem to have prevailed, at least during the first months into the pandemic. In addition, they provide further insights into the link between uncertainty and couple stability by exploring different dimensions and levels of uncertainty. First of all, despite showing a strong correlation with each other, the dimensions of employment and income uncertainty played independently a significant role in predicting the risk of couple dissolution. Furthermore, different levels of uncertainty affected the risk of separation to a different extent: while indicators of longer-term employment and income uncertainty were strong predictors of couple dissolution, changes in uncertainty occurred in the pandemic context were only partially associated with couple separation. Neither the loss of employment by the end of contract nor situations of partial activity were significantly associated with the risk of separating. This might be due to the fact that these situations of employment uncertainty were recent (and still of short duration), often compensated by employer or state specific measures, and less stigmatising in a context of crisis. Also, in situations of partial activity, the high level of protection of workers might have decreased the level of uncertainty and stress experienced. On the other hand, a deterioration in the financial situation declared at both waves was associated with a higher risk of separation throughout the considered time period, net of low household income before the beginning of the pandemic. Thus, while employment uncertainty seems to matter for couple stability rather when it is long-lasting and associated with stigma, income uncertainty can increase the risk of separation in the shorter term, and even at a time when it is widespread.

Our results highlight that it is important to investigate the potential effects of the Covid-19 crisis for family stability and well-being. High separation rates can have important implications for the lives of many families affected by the pandemic, not least on fertility rates. They also suggest that increases in income and employment uncertainty due to the pandemic crisis might be an important driver for couple separation, in line with what hypothesized by family stress theories (Schmid et al., 2021). Our results partly confirm the hypothesis of a “shocking” and immediate effect of the first lockdown, leading a relatively high proportion of couples, and especially those experiencing increased income uncertainty, or long-term employment uncertainty, to separate within the first months following the lockdown. The high levels of separation occurring shortly after the first lockdown suggest that the stress linked with income and employment uncertainty could have been accentuated because partners were obliged to spend together longer times than usual (because of lockdown), which might result in more tensions and conflict (Settersen et al., 2020). Moreover, individuals who experienced a persistent deterioration in financial situation after the first months of the pandemic crisis had higher risks of separating later, which means that the feeling of uncertainty may have also long-lasting effects. However, they also suggest that it was situations of longer-term employment uncertainty, rather than sudden unemployment linked with the pandemic, to be determinant for the risk of separation in the short run.

The substantially higher separation rates observed in the first six months after the lockdown compared to the following months might also be partly explained by selective effects: couples who were less solid at the beginning of the pandemic did not manage to face the stressful situation and separated just after the first lockdown. Couples that managed to live together during the first months of the pandemic were the more solid and thus less likely to separate afterwards. The long-lasting effects of income uncertainty may thus matter for both the more unstable or young couples who were able to separate quickly as well as those more committed who might take more time to separate or divorce. To check that our results of low separation rates in the second time period were not due to the selected profile of couples at risk, i.e. those who already survived the first lockdown together, we run our analyses on separations occurring between wave 2 and wave 3 considering also couples who were formed between wave 1 and wave 2. The results were very similar to the ones we showed above.

Income uncertainty predicts the risk of separation more for men than for women. The fact that declarations about the household financial situation provided by men play more on couple dissolution risk than the ones provided by women is puzzling and interesting. Although the proportions of men and women declaring worse financial situations are similar on average (it is not possible to compare within the couple unfortunately), this could suggest that men feel more responsible, worried or anxious by the household financial deterioration than women. In spite of widespread model of dual-earners couples, the mental workload of the financial household situation seems than to remain more on the men’s shoulders, as there is evidence that the mental workload of the domestic tasks remains on the women’s shoulders. Thus, conditional on objective employment and income situation, men’s worries or subjective feeling about finances plays more than women’s ones.

An important limitation of this study is that we did not have any information about quality of relationship of the couples, or on how long they had been together before being included in the survey, though the marital status is a (poor) proxy. It is therefore not clear whether the lockdown was the cause of the couple's crisis or whether it worsened a poor marital climate and accelerated the separation. However, to make sure that our findings were not driven by the high share of “frail unions”, we performed our analyses only considering those couples in Epicov who also were registered as couples by the fiscal data in 2018 (thus the same sub-sample used to calculate separation rates in the last rows of Table 1). Results from the analyses of the association between uncertainty and the risk of separations provided very similar results to the ones shown. In addition, some household re-arrangements might have been due to Covid-related reasons other than couple relationship. To limit this, we selected couples who were living in their usual dwelling at the moment of the first interview, which excluded respondents who joined their partner’s home to get through the lockdown together, as well as couples who decided to live elsewhere at the moment of lockdown. However, couples in the opposite situation- respondents whose partners moved in for the lockdown- were included in our analysis since we were not able to identify them. This would mean that our separation rates are to some extent over-estimated because they include part of these very recently formed and possibly more fragile couples. We can assume that the proportion of respondents who had the partner moving in for the lockdown was similar to the one of those who moved to their partner’s home, and then returned to the usual dwelling afterwards. We performed alternative models that include these couples where the respondent went to their partner's home to spend the lockdown, and results on the effect of employment and economic uncertainty were very similar to those previously obtained. Thus, the inclusion or exclusion of these frail unions does not seem to drive our results.

Another limitation, as already pointed out, is that we did not have information about the partner’s employment status. Couples where both spouses had to stop working may have experienced greater uncertainty. Finally, stop living together does not mean separation. Couples might decide to temporarily stop cohabiting, but will eventually get back together. Lastly, because we selected in our sample respondents who were interviewed during at least the first two waves, and that we can hypothesise attrition risk to be higher in case of dissolution, our estimations would be a low bound of real separations.^10^ However, contrary to housing-based surveys, Epicov survey was interviewing respondents by phone or Internet, which limits the attrition risk due to moving, whether the move is Covid-related or separation-related, which is a strength of this survey for studying events linked to moves such as separation. Also, to check that attrition throughout the survey did not affect too much our results, we run the analyses on the risk of separation between wave 1 and wave 2, only including individuals who were interviewed at all three waves. Again, results were virtually identical.

Nevertheless, the results of this study contribute to the existing literature by suggesting that situations of employment and income uncertainty are both associated with higher risks of separating, even in a context of generalised uncertainty such as the beginning of the Covid-19 pandemic. However, objective conditions do not necessarily matter: partial activity, that assured high levels of protection to the employees, or the end of a contract were not linked with higher risk of separations. In addition to government job protection measures that have guaranteed the return to employment, the exceptional situation of the pandemic, and especially the first strict lockdown, may have made unemployment less stigmatising than in normal times. Moreover, the recurrence of economic crises and the development of flexible forms of employment and the valorisation of job mobility in the new millennium may also make periods of unemployment more usual. Employment may also matter less for individuals’ identity in this context.

The unique experience of the lockdown highlighted the importance of financial conditions even in a context of large job protection such as France. We should expect deterioration of financial conditions to have even larger effects in countries with a less generous welfare. Future research will also need to investigate whether these results are specific to the exceptional situation of the pandemic, or whether they represent a more profound change in the relationship with employment, especially for men.

## Appendix

See Figs. 8, 9 and 10

## Footnotes

1 It fell by 2.5% in the first quarter, by 0.8% in the second, it rose by 1.6% in the third quarter, dropped by 0.1% in the fourth quarter, and then increased.

2 73.1% of two-person couples aged 30 to 54 are composed of a man and a woman are dual-earner in 2011.

3 Response probabilities were estimated using logit models adjusted for auxiliary variables potentially linked to both the response mechanism and the main variables of interest in the Epicov survey. The survey weight (the inverse of the inclusion probability) was divided by an estimate of the probability of response and then these weights were calibrated according to the margins of the population census data and population projections for several variables.

4 Separation rates by educational level are not shown because did not show significant differences.

5 Results from models considering separately variables concerning employment and financial uncertainty provided very similar results to those provided by the joint models.

6 As labour force surveys are based on the housing place and not individuals, they are not very efficient to measure separation rate since many separations involve a move of one or both partners. In that latter case, when all members of household change between two waves, we cannot distinguish a household move from a separation.

7 As the Epicov survey respondents were randomly chosen among the comprehensive fiscal data, there are some common variables.

8 Figures 8 and 9 in the Appendix show the individuals determinants of experiencing respectively a change in employment situation and in financial conditions.

9 This is confirmed by the fact that experiencing employment uncertainty during the lockdown is a strong predictor of declaring financial deterioration (Figure 9).

10 Younger, lower educated, lower income individuals and those who experienced changes in employment conditions were more likely not to be followed up at subsequent wave, and these characteristics are all predictors of couple separation.
